# Identifying a few foot-and-mouth disease virus signature nucleotide strings for computational genotyping

**DOI:** 10.1186/1471-2105-9-279

**Published:** 2008-06-13

**Authors:** Guohui Lin, Zhipeng Cai, Junfeng Wu, Xiu-Feng Wan, Lizhe Xu, Randy Goebel

**Affiliations:** 1 Department of Computing Science, University of Alberta. Edmonton, Alberta T6G 2E8, Canada; 2 Molecular Virology and Vaccine Branch, Influenza Division, Centers for Disease Prevention and Control. Atlanta, GA 30333, USA; 3 Animal and Plant Health Inspection Service, U.S. Department of Agriculture, Plum Island Animal Disease Center. PO Box 848. Greenport, NY 11944, USA

## Abstract

**Background:**

Serotypes of the Foot-and-Mouth disease viruses (FMDVs) were generally determined by biological experiments. The computational genotyping is not well studied even with the availability of whole viral genomes, due to uneven evolution among genes as well as frequent genetic recombination. Naively using sequence comparison for genotyping is only able to achieve a limited extent of success.

**Results:**

We used 129 FMDV strains with known serotype as training strains to select as many as 140 most serotype-specific nucleotide strings. We then constructed a linear-kernel Support Vector Machine classifier using these 140 strings. Under the leave-one-out cross validation scheme, this classifier was able to assign correct serotype to 127 of these 129 strains, achieving 98.45% accuracy. It also assigned serotype correctly to an independent test set of 83 other FMDV strains downloaded separately from NCBI GenBank.

**Conclusion:**

Computational genotyping is much faster and much cheaper than the wet-lab based biological experiments, upon the availability of the detailed molecular sequences. The high accuracy of our proposed method suggests the potential of utilizing a few signature nucleotide strings instead of whole genomes to determine the serotypes of novel FMDV strains.

## Background

Foot-and-mouth disease (FMD) is one of the most contagious animal diseases, with a large economic impact. Frequent sporadic outbreaks have been reported in many countries. The most recent outbreak in the United Kingdom costs tens of billions of dollars [1]. This disease causes extensive epidemics in domestic and wild cloven-hooved animals, such as cattle, sheep, goats, and pigs. In addition, it can result in persistent infections in ruminants, and so the disease is in the importation banning and detection list of most countries. Furthermore, this disease can cause mild infection in human through skin wounds or the oral mucosa. Therefore, once this disease is identified, the infected animals are always required to be destroyed. The vaccination is the most efficient method to prevent this disease, so preparation and selection of an efficient vaccine will be the most important for FMD prevention and control.

FMD is caused by a single strand RNA virus, so-called FMD virus [2], which is a member of the family Picornaviridae, genus *Aphthovirus*. The genome of FMDV is about 8.4 kbp in size (including highly structured 5' and 3' untranslated regions), and encodes 12 proteins – leader proteinase L^pro^, four structural proteins 1A (VP4), 1B (VP2), 1C (VP3), and 1D (VP1), and seven non-structural proteins 2A, 2B, 2C, 3A, 3B (VPg), 3C, and 3D [3]. Like other RNA viruses, mutation and recombination always facilitate the emergence of new FMDV strains. So far, seven immunologically distinct serotypes have been identified: Euroasiatic serotypes A, O, C, and Asia1 and South African Territories serotypes SAT1, SAT2, and SAT3. The capsid protein VP1 is exposed to the surface of the viron and contains serotype-specific amino acid sequence variations. Note that the dissimilarity in nucleic acid in the protein coding region among different serotypes can be up to 54% [3].

Traditional laboratory experiments for serotyping were based on the enzyme-linked immunosorbent assay (ELISA) [4,5] and the reverse transcriptase polymerase chain reaction (RT/PCR). More recent methods, such as antigen capture RT/PCR (Ag-RT/PCR) [6], employ type-specific antibodies (against immuno-reactive recombinant proteins) for virus capturing followed by RNA amplification. These methods generally target only the VP1 gene, or VP1 and other capsid-coding genes. They are very useful for selecting the correct vaccines in case of FMD outbreak, but they may not be able to identify the presence of new variants or recombinants of multiple serotype viruses, which are very common cases for FMD. However, identification of the presence of new variants or recombinants is essential for determining the source of outbreak, understanding the evolution of the virus, and advancing the FMDV epidemiological study. Recently, advances in genomic sequencing technologies allow us to obtain rapidly the complete genomes of FMDV, and this enhances the development and application of computational strategies in serotype and genotype analyses of FMDV [3,6-8]. Previous computational genotyping analysis is generally based on a multiple sequence alignment of the viral genomic sequences or their protein products [7]. For example, the most recent FMDV phylogenetic and recombination analysis used split decomposition [8] to examine the complete strains of 103 isolates [3]. However, this strategy is limited by data size (particularly, the number of sequences), that is, the larger the dataset, the lower accuracy can be achieved.

In this study, we propose to identify a set of signature nucleotide strings which can be readily used to efficiently detect the genotypes for emerging FMDV strains. Our method utilizes information theory and an advanced feature selection method to extract the serotype-specific nucleotide strings using the complete composition vector (CCV) representation [9] of a set of known FMDV strains and then to build linear-kernel support vector machines as a genotype classifier using these extracted strings. In addition to genotype analysis, these extracted signature strings may shed lights on virus evolution, especially within the unique regions in the viral proteins, which may be used for vaccine construction for virus recombinants.

## Results

The first FMDV dataset we collected contains in total 129 whole viral genomes [see Additional file 1 for their NCBI GenBank accession numbers]. This dataset is used for cross validation study. Among these 129 whole genomes, there are 47 serotype A strains, 48 serotype O strains, 8 serotype C strains, 9 serotype Asia1 strains, 9 serotype SAT1 strains, 4 serotype SAT2 strains, and 4 serotype SAT3 strains (Table 1, second column). The average length of these whole genomic sequences is 8, 151 bp (including highly structured 5' and 3' untranslated regions), with the maximum length 8, 280 bp and the minimum length 6, 996 bp. These FMDV sequences were downloaded from NCBI GenBank in December 2006. The second dataset contains 83 other FMDV strains [see Additional file 1 for their NCBI GenBank accession numbers], which were downloaded separately from NCBI GenBank in February 2007 and May 2008 and used for independent testing purpose. Their serotype composition is recorded in the 10th column of Table 1.

**Table 1:** LOOCV and independent genotyping accuracies

		A	O	C	Asia1	SAT1	SAT2	SAT3		A	O	C	Asia1	SAT1	SAT2	SAT3
	129	47	49	8	9	10	3	3	83	6	31	16	26	1	2	1
A	47	**47**							6	**6**						
O	48		**48**						31		**31**					
C	8			**8**					16			**16**				
Asia1	9				**9**				26				**26**			
SAT1	9					**9**			1					**1**		
SAT2	4		1				**3**		2						**2**	
SAT3	4					1		**3**	1							**1**

The composition of the different serotype FMDV strains in our two datasets (columns 2 and 10). The LOOCV genotype prediction results on the first dataset are in row 2 from columns 3 to 9 and more details in rows 3–9 and columns 3–9 as a confusion matrix; the independent testing results on the second dataset are in row 2 from columns 11–17 and more details in rows 3–9 and columns 11–17 as a confusion matrix. Numbers in bold denote the correct genotype predictions.

**Figure 1:**
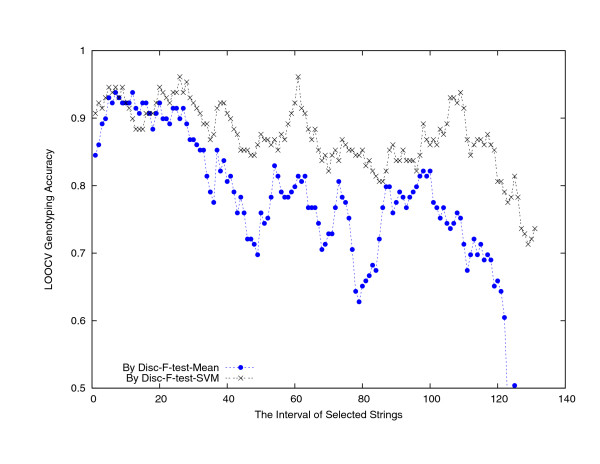
**The LOOCV genotype prediction accuracies of the SVM-classifier and the Mean-classifier using 20 consecutive nucleotide strings in the string list selected by the Disc-F-test method.** That is, the accuracies at position *k *are the ones by the SVM-classifier and the Mean-classifier built using strings *k *to *k *+ 19, respectively.

**Figure 2:**
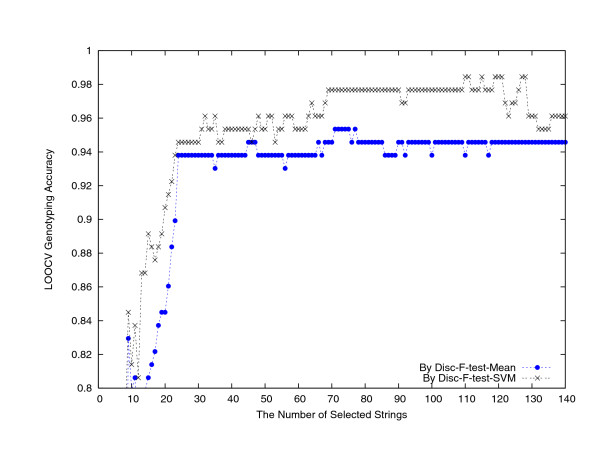
The LOOCV genotype prediction accuracies of the SVM-classifier and the Mean-classifier using the top ranked nucleotide strings by the Disc-F-test method.

Setting the maximum string length to 15, there are in total 1,320, 791 distinct nucleotide strings occurring in the first dataset of 129 FMDV strains. We computed the composition values for each of these strings in the 129 strains and retained the top 10, 000 ones ranked by *revised relative entropy *(RRE) [9,10]. By this point, the serotype information of the strains had not been used. These 10,000 top ranked strings have their length in between 4 and 11 and the detailed percentages are collected in Table 2, where length-6, 7, 8 strings show dominant (97.11%). In the next stage of signature nucleotide string identification and validation, we adopted the leave-one-out cross validation (LOOCV) scheme [11], in which at every run one strain was held out as testing sample while all the others, with their labeled serotype, form the training dataset. For string identification purpose, we adopted the biomarker identification method, the Disc-F-test method [12]. Essentially, the *k*-means algorithm was used to cluster these 10, 000 strings into 150 clusters, using their composition values across the 128 training strains and the differences between the mean composition values in different serotypes (which were used to define the Euclidean distance between two strings, see Methods and [12]); and the F-test method [13] was run to re-sort the 10,000 strings, incorporating the strain serotype information; then the Disc-F-test method walked through the string order by the F-test method to pick up one string per cluster. For extracted string validation purpose, we examined their power in genotyping the testing strain. That is, the strings selected by the Disc-F-test method were used to build an SVM-classifier and a Mean-classifier [14]. Applying a sliding window of width 20 along the Disc-F-test string list to use only 20 strings to build the two classifiers, their classification accuracies are plotted in Figure 1, respectively. From these two plots, one can see that the genotype recognition strength of strings selected by Disc-F-test was decreasing in general, and particularly lower than 80% after rank 120. Using the first *k *strings by the Disc-F-test method, for *k *= 1,2, 3,..., 140, the LOOCV accuracies of the SVM-classifier and the Mean-classifier are plotted in Figure 2. The highest LOOCV accuracy reached by the Mean-classifier was 123/129 = 95.35%, when using 71–75 or 77 selected strings; The highest LOOCV accuracy reached by the SVM-classifier was 127/129 = 98.45%, when using around 120 selected strings (Table 1 columns 3–9, where SAT2 strain AY593849 was predicted as O and SAT3 strain AY593850 was predicted as SAT1). Figure 2 shows a clear pattern that the Disc-F-test-SVM-classifier performed the best.

**Table 2:** Composition of the top ranked 10, 000 nucleotide strings by RREs

String Length	4	5	6	7	8	9	10	11
Percentage (%)	0.01	0.59	12.27	57.43	27.41	2.11	0.12	0.06

The percentages of different length nucleotide strings in the top ranked 10, 000 strings by their RREs.

**Figure 3:**
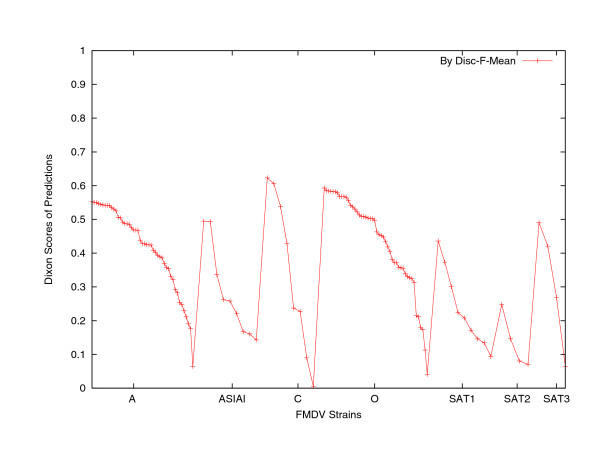
Confidence of the LOOCV genotype prediction of the Mean-classifier using the nucleotide strings selected by the Disc-F-test method.

The linear kernel SVM-classifier constructs seven decision hyperplanes in the high dimensional space that best separate each genotype from the others. Upon the arrival of the testing strain, it computes the distance between the testing strain, represented also as a vector in the high-dimensional space, and each of the hyperplanes. The serotype associated with the closest hyperplane to the testing strain is taken as the predicted serotype for the testing strain. During the experiments, we were unable to return all these distances for prediction confidence evaluation purpose. For the Mean-classifier, associated with the testing strain, let *d*_1 _and *d*_2 _denote the shortest and the second shortest average distances, respectively, and *d*_7 _denote the longest average distance between the testing strain and the seven serotypes. We calculated (*d*_2 _- *d*_1_)/(*d*_7 _- *d*_1_) (*Dixon metric*) [14] to be the quantified confidence associated with the prediction. For all the 129 testing strains, their prediction confidence values of the Disc-F-test-Mean-classifier, using the top ranked 120 nucleotide signature strings, are plotted in Figure 3 and partitioned into different genotypes and in non-increasing order. Only 8 of 129 predictions have confidence less than 0.1, which is the normal threshold for high confidence [14]. These 8 strains include 1 A strain, 2 C strains, 1 O strain, 1 SAT1 strain, 2 SAT2 strains, and 1 SAT3 strain. Among these 8 predictions, 4 were incorrect.

### Independent genotyping results

Using the first dataset of 129 FMDV strains as the training dataset, and applied the above procedure to firstly rank all occurring strings by RRE, then re-rank the top 10, 000 of them by F-test using the strain serotype information (assuming the serotypes of all the 129 strains are correct, see Discussion), and lastly select 140 strings using the Disc-F-test method. Notice that these 140 strings could slightly differ from each of the sets of 140 strings in the above LOOCV study. Using the linear-kernel SVM classifier built on them, every strain in the second dataset, 83 strains in total, was submitted to have its genotype predicted. We achieved 100% prediction accuracy in this independent test (Table 1, Columns 11–17).

## Discussion

### Always mis-typed strains

For each of the 129 viral genomes in the first dataset, we submitted it to BLAST and assigned its genotype using either the closest hit or a majority vote from 5 nearest neighbors. SAT1 strain AY593844 was incorrectly typed when using the closest hit, and four SAT2 strains AF540910, AY593847, AY593848, and AY593849 were mistyped using the second rule. We note that the genome database we used in BLAST contains many more FMDV strains than we have (most of these extra sequences are not complete genomes), yet BLAST made some unexpected mistakes.

The linear-kernel SVM classifier we constructed using 140 strings selected by RRE and Disc-F-test achieved 127/129 = 98.45% LOOCV genotyping accuracy and made mistakes on SAT2 strain AY593849 and SAT3 strain AY593850. One possible reason for these mistakes is the limited number of SAT isolates in our dataset, compared to Euroasiatic serotypes, which formed an unbalanced training dataset. On the other hand, similar to many other RNA viruses, FMDVs have been reported with active recombination, which may be located in not only the structural protein coding regions but also the non-structural protein coding regions [3]. Phylogenetic analyses have shown the incongruent topologies between the genes, for example, L^pro^, 3C^pro^, and 1D [3,15-18]. The above two mistyped strains by our SVM classifier have been reported with potential recombination [3]: strains AY593849 (SAT2/3 Kenya 11/60) and AY593850 (SAT3/2 SA57/59) were reported with conflicting phylogenetic topologies over the overall genomic sequences and different regions (*e.g*., L^pro^/2A to 3D). We also examined these two mistyped strains by analyzing the different fragments on their genomes using our SVM classifier. The region-by-region analyses revealed that their P1 region, especially the VP1 gene which is used currently for the FMDV serotyping, is closely related to their GenBank recorded serotype. However, the other different regions in their genomes are closer to the genotypes predicted by our SVM classifier than to their recorded serotypes. In fact, the top 10 strings we used for genotyping are all outside VP1 region (CCGCCTG, TAAGGTA, AGTCCAT, TTCATCAA, ACCGACGG, CCAGTGAA, GCGACAAC, ACCAACAT, GTTTCT, CACATGG), which further support our prediction method. For example, VP2, VP3 and VP1 genes in strain AY593850 show high sequence similarities with isolates of SAT3; While its P2, P3 and 3' UTR regions share more sequence similarities with SAT1 and SAT2 strains, rather than with SAT3 strains. For strain AY593849, a BLAST search using its full genomic sequence showed that the top 5 statistically significant hits are one SAT2 strain, one SAT1 strain, and three Asia1 strains, which was unexpected since there were several other SAT2 strains in GenBank. Therefore, we strongly believe that the mistyped results were caused by potential recombination between FMDVs. Future study will be to identify the potential recombination cases using the signature string information.

### The maximum string length

In our experiments, we set the maximum string length to 15. The rationale on setting this value was that increasing it did not improve the genotyping accuracy, since essentially strings of length greater than 11 did not make into the list of top ranked 10, 000 strings by RRE. Nevertheless, long serotype-specific amino acid motifs have been proposed/discovered, for example, YSTXEDHXXGPN was believed serotype A specific, YXTXEDFVXGPN was believed serotype O specific, YATXEDXXGPN was believed serotype C specific, YXVXEDAVSGPN was believed serotype Asia1 specific, YAXXDXFLPGPN was believed serotype SAT1 specific, YADXDSFRXGPN was believed serotype SAT2 specific, and YXSADRFLPGPN was believed serotype SAT3 specific [3]. We have therefore increased the maximum string length to 26 (The experiment failed on the maximum length 27 due to insufficient computer memory. All these experiments were carried out on a Heisler cluster node with a 2.2 Ghz CPU and 5.0 GB memory.) and applied all the above described genotyping methods. Again, we found no improvement on genotyping accuracy, and only two length-16 strings were able to make into the top 10, 000 strings though still not selected by either the F-test or the Disc-F-test method.

### The size of training dataset

**Figure 4:**
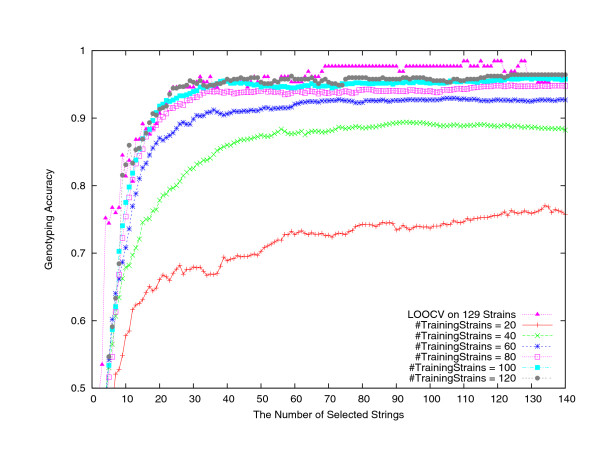
A Monte Carlo type analysis on the average genotyping accuracy over 100 independent testing datasets of size 129 - *n*, corresponding to the training datasets of different size *n*, for *n *= 20, 40, 60, 80, 100, 120.

On the first dataset of 129 strains downloaded in December 2006, we have conducted the LOOCV study. This first dataset was also used as the training dataset to genotype the second dataset of 83 strains which were downloaded from NCBI GenBank in February 2007 and May 2008 separately. Both the LOOCV and the independent testing show promising computational genotyping results. One may question how large a training dataset should be in order for effective genotyping. We have conducted a Monte Carlo type analysis to form training subsets of *n *randomly chosen strains from this first dataset of 129 strains, for *n *= 20, 40,60, 80, 100, 120. For each value of *n*, 100 random subsets were formed. On each such subset the same informative string selection was performed and subsequently the SVM-classifier was built. The strains not in training dataset, 129 - *n *strains in total, formed the independent testing dataset, on which the genotyping accuracy was collected. The average genotyping accuracy over 100 datasets of the same size *n *is plotted in Figure 4, which clearly shows that for effective genotyping a substantial number, such as 80, of training strains is necessary. Note that this is nevertheless understandable given that there are 7 subtypes of FMDVs. The LOOCV genotyping result on the first dataset of 129 strains is also plotted in Figure 4, which matches nicely with the result using 100 or 120 training strains (or roughly the 5–10-fold cross validation [11] result on the set of 129 strains).

### Other experimental settings

We have also experimented with the 20, 000 top ranked strings by RRE and found that these 20, 000 strings have their length in between 4 and 15 (the detailed percentages not shown) and length-6, 7, 8 strings are still dominant (93.445%). Applying the same string selection methods coupled with the SVM-and Mean-classifiers (built on up to 140 strings) did not improve the genotyping accuracy, but decreased a little. The highest accuracy observed was 125/129 = 96.90%, by Disc-F-test-SVM-classifier, when selecting 100 strings. Moreover, the top 20 strings selected by Disc-F-test from the 20, 000 strings shared 10 strings in common with the top 20 strings selected by Disc-F-test from the 10, 000 strings, and their ranks were roughly the same (ranked 1–5 and 7–11). This fact also suggested that these 10 common strings should carry rich serotype specific information.

In addition to the above string composition values computed within the Markov model [9,19,20], we have also examined the genotyping performance using the string occurrence frequencies. It turned out that using the composition values performed significantly better than using the occurrence frequencies (data not shown). Several other advanced feature selection methods and another well-known *k *nearest neighbors (KNN) classifier have also been examined (results not shown). The results were all compatible though slightly worse, indicating that computational genotyping FMDV strains via a few serotype-specific nucleotide strings is feasible and can be successful.

### Other computational genotyping results

We have also conducted several other ways of whole genome based computational genotyping on the FMDV strains. For example, we constructed a multiple sequence alignment for the 129 strains, via ClustalW (which took 18.7 hours on a desktop with a 2.1 GHz CPU and 2.0 GB memory to complete), and the associated phylogenetic tree [see Additional file 2] showed at least the two misplaced strains, SAT1 strain AY593844 and SAT2 strain AY593849. In fact, this is the only result that is about equally good to our Disc-F-test-SVM classifier, the latter took only seconds to type all the 129 strains under the LOOCV scheme.

Along the CCV-representation approach, using the top 10, 000 nucleotide strings ranked by RRE, every strain was represented as a 10, 000-dimensional vector. Applying the standard *principle component analysis *(PCA) on this 10, 000 *× *129 matrix to obtain the first two principle components (PCs), the *linear discrimination analysis *(LDA) showed that all the Euroasiatic serotype strains well separated from those South African Territories (SAT1-3) strains, except SAT1 strain AY593844 and SAT2 strain AY593849 which appear close to Euroasiatic strains [see Additional file 3]. Within Euroasiatic serotype strains, some of them, for example, some serotype O strains, showed large distances from the other strains, while some others seemed to mix up together. Within SAT serotypes, the strains all mixed together and were seemingly inseparable. Increasing the number of PCs (up to 9) could obtain some finer resolution results but the general conclusions remained the same (data not shown). This indicates that CCV-PCA-LDA might not be a good approach for FMDV genotyping, though PCA has been successful in many other applications.

Note that it is computationally impossible to perform PCA on all the 1, 320, 791 strings. Nevertheless, we were able to use the 1, 320, 791-dimensional representation vectors to calculate the pairwise distances for all the 129 strains, and subsequently submit them to Neighbor-Joining method in Phylip 3.70 to construct a phylogeny [see Additional file 4]. This phylogeny showed that, though common serotype strains were largely clustered into separate clades, there were at least 7 misplacements. Applying the same procedure but using only the 10, 000 top ranked strings, the Neighbor-Joining tree showed at least 9 misplacements [see Additional file 5].

During the LOOCV scheme for selected string validation, we have also tested distance-based genotyping approach. Using all 1, 320, 791 strings on the training dataset, we have calculated the Euclidean distance from the testing strain to each of the 128 training strains, then calculated the average distance from the testing strain to each of the 7 serotypes, and finally assigned the testing strain with the closest serotype. The LOOCV accuracy of such a Mean-classifier was 123/129 = 95.35%. We repeated this process using only the 10, 000 top ranked strings by RRE, and the LOOCV accuracy of the corresponding Mean-classifier was 108/129 = 84.72%. Using all 1, 320, 791 strings and the 10, 000 top ranked strings, respectively, linear-kernel SVM classifiers were also built and used to predict the genotype of the testing strain. The LOOCV accuracies of these two SVM-classifiers were both 120/129 = 93.02%.

Using only the *k *top ranked nucleotide strings, for *k *= 1, 2, 3, ..., 140, by RRE, we have also collected the LOOCV accuracies of the Mean-classifier and the SVM-classifier [see Additional file 6]. The highest accuracy by the Mean-classifier and the SVM-classifier were 111/129 = 86.05% and 118/129 = 91.47%, respectively. The general tendency was that the Mean-classifier performed slightly worse than the SVM-classifier. Among the 10, 000 top ranked strings by RRE, using the *k *top ranked strings by the F-test method, for *k *= 1, 2, 3,..., 140, the LOOCV accuracy of the SVM-classifier has been collected [see Additional file 7, labeled with F-test-SVM-classifier]. Typically, when *k *= 140, the LOOCV accuracy reached 123/129 = 95.35% (the highest by F-test-SVM-classifier). The LOOCV accuracy of the Mean-classifier using these *k *strings has also been collected [see Additional file 7, labeled with F-test-Mean-classifier]. Typically, when *k *= 140, the LOOCV accuracy reached 124/129 = 96.12% (the highest by the F-test based classifiers).

## Conclusion

We proposed a method to select the most informative and serotype-specific composition nucleotide strings for FMDV whole genome genotyping. Such a proposal appears novel in the context of FMDV genotyping, with at least three advantages: 1) It does not involve the high complexity stage of multiple sequence alignments, and thus supports high throughput genotyping. This simplifies the genotyping process through sequencing information and thus shortens the disease control process – once the genotype is defined, the decision on the type of vaccine can be made. Therefore, it helps to determine the source of FMDV in case of an outbreak. The potential ability to recognize a recombinant, in addition to genotype FMDV, makes our method very valuable, especially for wars against bio-terrorists. 2) It considers the whole genomic sequences for genotyping, and filters potential random mutation at the same time. Therefore, it provides an additional and/or complementary genotyping tool to the current available methods. Moreover, computational genotyping is much faster and much cheaper than the wet-lab based biological experiments, upon the availability of the detailed molecular sequences. Note that the whole process including computing the genome CCV representation, the LOOCV scheme to select signature nucleotide strings, to build classifiers subsequently, and to predict the serotypes for testing strains, took less than 10 minutes on our first dataset. Excluding the training process, genotyping one testing strain took less than one second. In this sense, the proposed computational genotyping is much faster and less expensive than the wet-lab-based FMDV serotyping. 3) It adopts feature selection methods to identify composition nucleotide strings that are the most serotype-specific, and thus allows biological explanation on the genotyping results. The identified signature strings may also facilitate the preparation of recombination vaccine. It is interesting, but not completely unexpected, to see that using only around 120 strings selected by the Disc-F-test method, the genotyping accuracy on the set of 129 FMDV whole genomes by the SVM-classifier can reach as high as 98.45%.

## Methods

The first FMDV dataset we downloaded in December 2006 from NCBI GenBank contains in total 129 whole viral genomes, among which there are 47 serotype A strains, 48 serotype O strains, 8 serotype C strains, 9 serotype Asia1 strains, 9 serotype SAT1 strains, 4 serotype SAT2 strains, and 4 serotype SAT3 strains (Table 1, second column). The second dataset contains 83 other strains, which were downloaded separately in February 2007 and May 2008 from NCBI GenBank and used for independent testing. Their serotype composition is recorded in the 10th column of Table 1.

For each nucleotide string of length from 1 to 15, we calculated its composition value [19] in every FMDV strain, and subsequently represented FMDV strains using their *complete composition vectors *[9]. In more details, given a viral strain *G *of length *L*, the number of appearances of a length-*k *nucleotide string *α*= *a*_1_*a*_2 _...*a*_k _in *G*, where every *a*_*i *_is a nucleotide, is denoted as *f*(*α*). Since there are *L - k *+ 1 (overlapping) length-*k *nucleotide strings in *G *in total, the frequency of appearance of string *α *in strain *G *is *p*(*α*) = *f*(*α*)/(*L - k *+ 1). Based on all these nucleotide string appearance frequencies, we can calculate the composition value *π*(*α*) for string *α *[19]. That is, we first calculate the expected appearance frequency of string *α *= *a*_1_*a*_2 _...*a*_*k *_as *q*(*a*_1_*a*_2 _... *a*_*k*_) = $\frac{p(a_{1}a_{2}\ldots a_{k-1})\times p(a_{2}a_{3}\ldots a_{k})}{p(a_{2}a_{3}\ldots a_{k-1})}$, and then define $\pi (\alpha )=\frac{p(\alpha )-q(\alpha )}{q(\alpha )}$. All these nucleotide string composition values are stored in sequence, to form a vector *V*_*k*_(*G*) = ⟨*π*_1_, *π*_2_,...,*π*_*m*_⟩ that represents the viral strain *G*, where *k *is the nucleotide string length and *m *denotes the total number of length-*k *nucleotide strings under consideration. In this work, we examine all the nucleotide strings of length from 1 to 15 (the reason for setting the maximum string length to 15 is provided in Discussion). The vector definition by using all these composition values of nucleotide strings of length from 1 to 15, i.e., the concatenation of *V*_1_,*V*_2_, ...,*V*_15_, is referred to as the *complete composition vector *of the viral strain. We note that from the definition, a maximum string length larger than 15 might be able to provide a vector containing finer evolutionary information, yet our empirical studies showed that 15 is probably large enough (see Discussion). We also want to point out that such a whole genome representation extends nucleotide composition vector and has been used and justified in biological sequence analysis for a long time [9,19-22]. Theoretically, there are 4 + 4^2 ^+ 4^3 ^+ ... + 4^15 ^= 1, 431, 721, 300 nucleotide strings need to be considered, but it turned out that only a fraction of 1,320, 791 strings whose composition values need to be calculated for our collected datasets. We implemented a prefix tree for the task.

Next, we concatenated all the 129 strains into a super-strain and similarly compute its complete composition vector. Using this vector as the background, we borrowed the concept of *relative entropy*, also known as *Kullback-Leibler distance*, to define a *revised relative entropy *(RRE) [9,10] measuring the distance between the series of composition values of a nucleotide string in the 129 strains and the background. Such an RRE value is expected to estimate the information content of strings across the FMDV strains, and the larger the absolute RRE, the more informative a string is. We kept only the top 10, 000 ranked strings (by their RREs). Two interesting facts about the FMDV dataset were that these top ranked 10, 000 strings have length less than or equal to 11, and that 97.11% of them have length 6, 7, or 8. Such facts on one hand confirm partially the decision that we can skip longer strings in the analysis, on the other hand, support the observation that using a single string length, as is done in [19,20], is not sufficient [9].

Using this 10, 000-dimensional vector representation for FMDV strains, the euclidean distance between every pair of strains can be calculated. These pairwise distances treated all 10, 000 nucleotide strings equally, in terms of their potential contribution in genotyping. A better way would be using these strings as features to classify the FMDV strains into different serotypes. However, the large gap between 10,000 features and 129 FMDV strains could result in non-unique classifiers which would be significantly biased on these 129 strains. Our next step was to further select a smaller number of strings out of the 10, 000 to build an effective serotype predictor for novel strains. To do this, each strain is represented as a 10, 001-dimensional vector, in which the last entry records the serotype label. We then applied one of the most effective feature extraction methods, the Disc-F-test method [12], developed within our group originally for identifying human cancer biomarkers using microarray gene expression data, to select 140 most discriminative strings. Essentially, this Disc-F-test method regards each string as a gene, the composition value as its expression value, and the strains as microarray samples. Under the *leave-one-out cross validation *(LOOCV) scheme [11], it first applies the F-test method [13] to re-order the 10,000 strings, using their composition values in 128 of the 129 strains (called *training samples*; the other strain is held out for testing purpose, the *testing sample*, whose serotype label is blinded to the constructed predictor). A string receives a high score if its composition values are close to each other in strains of the same serotype, but distinct to each other in strains of different serotypes. At the same time, the Disc-F-test method uses a *k*-means algorithm to cluster the 10, 000 strings into 150 clusters, using again their composition values in all the 128 training strains, and additionally the differences between their mean composition values in different serotypes. The method then walks through the string order determined by the F-test method to pick up one string per cluster for the first 140 clusters. Note that setting up 10 more clusters in the *k*-means clustering algorithm was to put away some strings that are not directly useful for genotype classification. These 140 selected strings, together with their composition values in all the 128 strains, were fed into a linear kernel SVM to build a classifier. Later on, for the testing strain, the composition values for only these 140 strings were calculated and such a 140-dimensional vector was sent to the SVM-classifier for genotype prediction.

## Authors' contributions

GL conceived the study and participated in its design, data collection, analysis, and manuscript preparation. ZC, XFW, LX, and RG participated in data collection, result analysis, discussion, and manuscript preparation. JW wrote the first set of CCV codes. ZC wrote the second version of the CCV codes and conducted all the computational experiments. All authors read and approved the final manuscript.

## Supplementary Material

Additional file 1 The FMDV strains used in this paper. This lists the NCBI GenBank accession numbers of the 129 FMDV strains in the first dataset and the 83 FMDV strains in the second independent testing dataset. Click here for file

Additional file 2 The MSA tree on the 129 FMDV strains. This MSA tree is constructed on the 129 FMDV strains using their whole viral genomes. In this tree, two SAT strains are misplaced. Click here for file

Additional file 3 The PCA analysis on the 129 FMDV strains. Two component LDA is done using the first two PCs from PCA on the 129 strains each represented as a 10, 000-dimensional vector. Click here for file

Additional file 4 The Neighbor-Joining tree on the 129 FMDV strains. This Neighbor-Joining tree is constructed on the 129 FMDV strains each represented as a 1, 320, 791-dimensional vector. In this tree, again, Euroasiatic strains and SAT strains are well separated, but they internally seem to mix up. Click here for file

Additional file 5 The Neighbor-Joining tree on the 129 FMDV strains. This Neighbor-Joining tree is constructed on the 129 FMDV strains each represented as a 10, 000-dimensional vector. In this tree, again, Euroasiatic strains and SAT strains are well separated, but they internally seem to mix up. Click here for file

Additional file 6 The LOOCV genotype prediction accuracies. This plots the LOOCV genotype prediction accuracies of the SVM-classifier and the Mean-Classifier using the top ranked strings by RRE. Click here for file

Additional file 7 The LOOCV genotype prediction accuracies. This plots the LOOCV genotype prediction accuracies of the SVM-classifier and the Mean-classifier using the top ranked strings by the F-test method and the Disc-F-test method. Note that Figure 2 in the main text is a portion of this figure. Click here for file
